# Comprehensive Characterization of a Cluster of Mucopolysaccharidosis IIIB in Ecuador

**DOI:** 10.3390/diagnostics15182337

**Published:** 2025-09-15

**Authors:** María Lucía Castro Moreira, Yorran Hardman Araújo Montenegro, Angélica Salatino-Oliveira, Héctor Quintero Montano, Rodolfo F. Niz Bareiro, Simone Silva dos Santos-Lopes, Thiago Ramos da Silva, Lucas Kelvy Sales Azevedo, Karyme Beatrice Lourenço da Silva, Affonso Weslley de Almeida Moreira, Suzany Silva Araujo, Francyne Kubaski, Franciele Barbosa Trapp, Ana Carolina Brusius-Facchin, Fernanda Medeiros Sebastião, Kristiane Michelin-Tirelli, Guilherme Baldo, Roberto Giugliani, Durval Palhares

**Affiliations:** 1 Faculdade de Medicina, Universidade Federal do Mato Grosso do Sul, Campo Grande 79070-900, MS, Brazil; luciacastro61@hotmail.com (M.L.C.M.); palharesdb@gmail.com (D.P.); 2 Hospital de Clínicas de Porto Alegre, Porto Alegre 90035-903, RS, Brazil; yorran_montenegro@hotmail.com (Y.H.A.M.); geli.oliveira@gmail.com (A.S.-O.); ftrapp@hcpa.edu.br (F.B.T.); afacchin@hcpa.edu.br (A.C.B.-F.); fsebastiao@hcpa.edu.br (F.M.S.); ktirelli@hcpa.edu.br (K.M.-T.); gbaldo@hcpa.edu.br (G.B.); 3 Facultad Ciencias de la Salud, Univesidad Laica Eloy Alfaro, Manta 27W3+MP, Manabí, Ecuador; hquintero@hotmail.com; 4 Hospital Universitário Maria Aparecida Pedrossia (HUMAP), Universidade Federal do Mato Grosso do Sul, Campo Grande 79070-900, MS, Brazil; rodolfobareiro@yahoo.com.br; 5 Biology Departament, Universidade Estadual da Paraíba, Campina Grande 58429-500, PB, Brazil; simonelopes@servidor.uepb.edu.br (S.S.d.S.-L.); thiago.silva@aluno.uepb.edu.br (T.R.d.S.); lucas.azevedo@aluno.uepb.edu.br (L.K.S.A.); karyme.silva@aluno.uepb.edu.br (K.B.L.d.S.); affonso.moreira@aluno.uepb.edu.br (A.W.d.A.M.); suzany.araujo@aluno.uepb.edu.br (S.S.A.); 6 Greenwood Genetic Center, Greenwood, SC 29646, USA; fkubaski@udel.edu; 7 Casa dos Raros, Porto Alegre 90610-261, RS, Brazil; 8 Centro de Ciências da Saúde, Universidade Federal do Rio Grande do Sul, Porto Alegre 90035-003, RS, Brazil; 9 DASA Genômica, São Paulo 05452-030, SP, Brazil

**Keywords:** Ecuador, ancestry, lysosomal storage diseases, mucopolysaccharidosis III, Sanfilippo syndrome

## Abstract

**Background/Objectives:** Sanfilippo Syndrome type B or Mucopolysaccharidosis type IIIB (MPS IIIB, OMIM 252920) is a lysosomal storage disease caused by deficiency of alpha-N-acetylglucosaminidase (NAGLU, E.C. 3.2.1.50) due to pathogenic variants in the *NAGLU* gene (17q21.2). The disease is characterized by progressive neurological manifestations, marked by cognitive decline, with relatively mild somatic involvement. We aim to present relevant information on a cluster of MPS IIIB identified in Ecuador, particularly regarding their clinical, biochemical, genetic, demographic, and ancestry characteristics. **Methods:** We present a characterization of a clinical, biochemical, genetic and demographic cluster of MPS IIIB patients in Ecuador, located in four main regions: Manabí, Guayas, Los Ríos, and Santo Domingo de los Tsáchilas. The patients included were diagnosed due to increased levels of urinary glycosaminoglycans (uGAG), plus deficient activity of NAGLU, and/or identification of biallelic pathogenic mutations in the *NAGLU* gene. Patients’ charts were reviewed for biochemical findings, medical history, clinical manifestations and assessments. **Results:** We present the results of clinical, biochemical, genetic and demographic characterization of a cluster in Ecuador with 24 patients identified with Sanfilippo syndrome type IIIB, resulting in an estimated incidence of 1.5/100,000. The mean age at diagnosis was 8.8 years, with symptom onset at 4.5 years on average. All patients exhibited elevated levels of uGAG and undetectable NAGLU activity, and all of them presented the c.1487T>C (p.Leu496Pro) variant in the *NAGLU* gene in homozygosis, indicating a possible founder effect, with the exception of one heterozygous one (p.Leu496Pro/p.Arg482Gln). A positive correlation between age of diagnosis and the concentration of one isoform of heparan sulfate (HS-OS) was found (*p* < 0.05). Clinical findings included neuropsychomotor developmental delay (75%), neurological regression (65%), hepatomegaly (55%), growth deficiency (50%), coarse facies (45%) and hernia (40%). Male patients presented earlier onset of symptoms. Maternal ancestry was successfully determined for 21 of the 24 patients. The majority were of Native American ancestry (71.4%), followed by European (19%), African (4.8%), and Asian (4.8%) lineages. Haplogroup A was the most prevalent (42.9%), followed by haplogroups D (19%), C, U, and H (each 9.5%), and R and L2 (each 4.8%). **Conclusions:** Ancestry can indicate a possible mechanism to explain the heterogeneous symptomatic presentation. These findings highlight the need for further research on genetic and environmental influences on disease severity in this population.

## 1. Introduction

Mucopolysaccharidosis type IIIB or Sanfilippo syndrome type IIIB (OMIM #252920) is a lysosomal storage disease caused by the presence of biallelic pathogenic variants in the *NAGLU* gene (17q21.2), resulting in a deficient enzymatic activity of alpha-N-acetylglucosaminidase (NAGLU, E.C. 3.2.1.50). The enzyme is involved in the degradation of glycosaminoglycans (GAGs) [1]. NAGLU deficiency causes the accumulation of heparan sulfate, which generates clinical presentations in affected patients, especially in the central nervous system [2]. The global incidence of the condition is estimated to be around 0.52:100,000 live births [3]. In Latin America there are few reports on the incidence of MPS IIIB, with previous studies in Brazil indicating 0.12:100,000 live births [4]. 

According to the literature, symptomatic presentation in MPS IIIB patients can be divided into three main phases. First phase usually occurs between 2 and 6 years of age, characterized by neuropsychomotor delay and behavioral problems i.e., aggressive behavior, agitation, and irritability [1,5,6,7,8]. During this phase, it is common to label the cases as “Autism Spectrum Disorder” [2]. The second phase occurs between 7 and 10 years of age, particularly due to progressive neurological regression and ineffective drug treatment for the abnormal behavior [5,6,7,8]. The third and final phase occurs after the first decade of life. Behavioral problems decrease significantly, and a severe neurodegenerative condition becomes well established [8]. Clinical progression of the disease continues until the second and third decades of life, leading to early death [1]. To date, there is no approved treatment for the disease, although therapeutic options are expected to become available in the coming years [9]. Given this scenario, diagnosis at the onset of symptomatic presentation will be essential for managing the patient’s quality of life.

Access to a correct diagnosis is one of the greatest challenges faced by patients and their families. Since 2005, the MPS Brazil Network has supported numerous physicians in Brazil and Latin America by providing biochemical and genetic tests using advanced methods such as tandem mass spectrometry and NGS panels at its laboratory [10,11]. Over the years, more than 1500 MPS diagnoses have been made, highlighting the significance of this initiative in the region [12]. By expanding our diagnostic support to other countries, we have identified several MPS clusters in Brazil and Latin America. In this study, we present relevant information on a cluster of MPS IIIB identified in Ecuador, focusing on their clinical, biochemical, genetic, demographic, and ancestry characteristics.

## 2. Methods

### 2.1. Clinical, Molecular, Biochemical and Demographic Characterization

**Figure 1:**
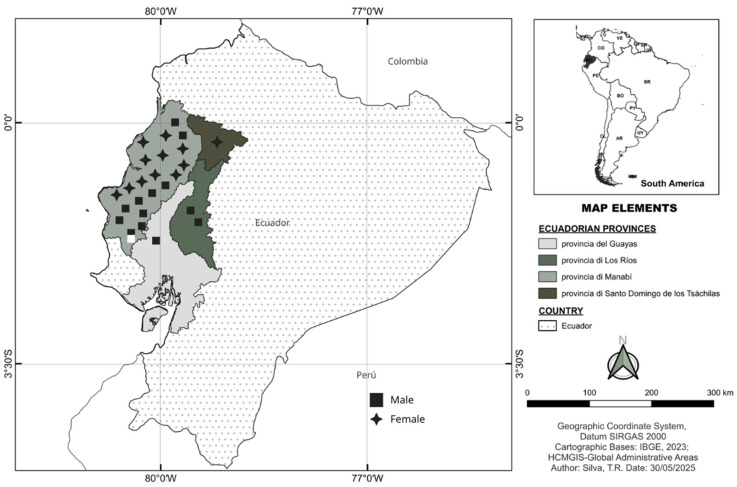
Map of Ecuador’s regions and birthplaces of MPS IIIB patients. Symbols in black indicate patients homozygous for the p.Leu496Pro variant. The white symbol is a compound heterozygous.

We present the characterization of clinical, biochemical, genetic and demographic clusters of MPS IIIB patients in Ecuador, located in four main regions: Manabí, Guayas, Los Ríos, and Santo Domingo de los Tsáchilas (Figure 1). The patients included were diagnosed due to increased levels of uGAGs plus deficient NAGLU activity, and/or the identification of biallelic pathogenic mutations in the *NAGLU* gene. NAGLU enzyme activity was measured from a dry blood spot on filter paper [10]. Molecular genetic analysis of the *NAGLU* gene was performed on DNA extracted from a dry blood spot on filter paper using next-generation sequencing on the Ion Torrent S5 platform, employing a pre-validated NGS panel [11]. Patients’ charts were reviewed for biochemical findings, medical history, clinical manifestations and assessments. The study was approved by the ethics committee from the Hospital de Clínicas de Porto Alegre (GPPG: 03-066, CAAE 60311622.7.0000.5327, 14 February 2003). Written informed consent was acquired from a parent for children or from patients over 18 years.

### 2.2. Structural Data

Structural coordinates of NAGLU protein (PDB: 4XWH) at 2.32 Å were retrieved from the Protein Data Bank. Information regarding variants was extracted from the NGS panel of MPS IIIB patients and modeled via i-tasser [13]. Each system was protonated at pH 7.0 using the PROPKA algorithm through the PDB2PQR web server [14]. The protein structure was visualized and analyzed using PyMol v. 2.3.0.

### 2.3. Molecular Analysis of Maternal Ancestry

Genomic material from the 21 samples was extracted using the blood spots protocol of the PureLink™ Genomic DNA Mini Kit (Invitrogen™, Thermo Fisher Scientific, Waltham, MA 02451, USA) following the manufacturer’s recommendations, with subsequent quality assessment by spectrophotometry (L-QUANT2, LOCCUS Biotechnology, Cotia, São Paulo, BR, Brazil) and precise quantification by fluorometry (Qubit 3.0, Thermo Fisher Scientific, Waltham, MA 02451, USA).

Maternal inheritance analysis (mitochondrial DNA) was performed by amplification and sequencing of hypervariable regions I (*HVS-I*), II and III (*HVS-II/III*) [15]. For the *HV-I* region, oligonucleotide primers L15997 (forward: 5′-CAC CAT TAG CAC CCA AAG CT-3′) and H017 (reverse: 5′-CCC GTG AGT GGT TAA TAG GGT-3′) were used, generating a 588 bp fragment. Amplification of the *HV-II/III* region was performed with primers L034 (forward: 5′-CCA TGC ATT TGG TAT TTT CG-3′) and H629 (reverse: 5′-TTT GTT TAT GGG GTG ATG TGA-3′), producing a 607 bp fragment. Amplification reactions were carried out in a final volume of 25 μL, containing 2 μL of genomic DNA (0.06–0.34 ng/μL), 18.5 μL of ddH_2_O, 0.5 μL of Sinapse^®^ Taq polymerase (0.1 U/μL), 2.5 μL of Sinapse^®^ reaction buffer (1×), 0.5 μL of dNTP Mix (0.05 mM) and 0.5 μL of each IDT^®^ primer (0.2 mM). Amplification was performed in a SimpliAmp™ thermocycler (Applied Biosystems, Thermo Fisher Scientific, Waltham, MA 02451, USA) with the following program: initial denaturation at 95 °C for 5 min; 35 cycles of denaturation (95 °C/30 s), annealing (55 °C/90 s), and extension (72 °C/30 s); followed by a final extension at 68 °C for 10 min. After amplification, 5 μL of each PCR product was subjected to electrophoresis on a 2% agarose gel prepared with 2 g of agarose, 100 mL of 0.5× TAE buffer, 5 μL of Sybr Green^®^ (Invitrogen™, Thermo Fisher Scientific, Waltham, MA 02451, USA), 5 μL of 2× TA, and 5 μL of Invitrogen^®^ 100bp Ladder (molecular weight marker) at 80 V for 40 min. Fragments were analyzed to confirm the expected sizes. The PCR products were purified using a combination of exonuclease I (EXO I) and shrimp alkaline phosphatase (SAP). The purified fragments were then subjected to a sequencing reaction using the BigDye™ Terminator v3.1 kit (Applied Biosystems) with the following thermal profile: initial denaturation at 96 °C for 1 min, followed by 40 cycles of denaturation at 96 °C for 15 s, annealing at 50 °C for 15 s, and extension at 60 °C for 4 min. After the sequencing reaction, the products were precipitated with a solution containing EDTA, sodium acetate, and absolute ethanol. Finally, bidirectional sequencing was performed on an automated ABI PRISM^®^ 3500xl Genetic Analyzer system (Thermo Fisher Scientific). Sequence alignment was performed using BioEdit v7.2 (biological sequence editor) and MEGA 11.0 (molecular evolutionary genetics analysis) [16]. Contigs were analyzed using the MITOMASTER platform [17] to identify mitochondrial polymorphisms. In parallel, the sequences were submitted to Haplogrep 3.2.1 [18], using the most updated database (rCRS—PhyloTree Build 17—Forensic Update 1.2) to determine haplogroups and haplotypes, allowing the inference of maternal ancestry. For validation, the sequences were compared with the Revised Cambridge Reference Sequence (rCRS; NC_012920.1) in BLAST-NCBI v. 2.17.0, with individual verification of each polymorphism identified in Haplogrep to confirm its presence in the analyzed sequences.

### 2.4. Statistical Analysis

Data were entered into a Microsoft Excel spreadsheet. SPSS version 12.0 for Windows (SPSS Inc., Chicago, IL, USA) was used for statistical analysis. Results are shown as descriptive data. Correlation between two variables was performed by Pearson’s correlation test. Multivariate Analysis was used for the descriptors, after they were transformed into binomial quantitative data. A *p* < 0.05 was considered significant.

## 3. Results

### 3.1. Cluster Characterization

**Figure 2:**
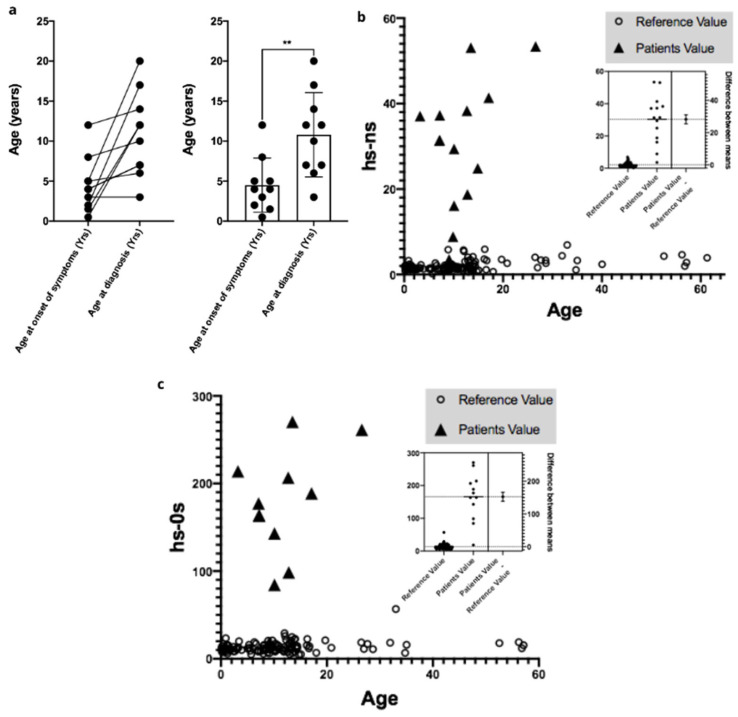
Age and biochemical results from MPS IIIB patients. (**a**) Age of onset of first symptoms and age of diagnosis. (**b**) Heparan sulfate Hs-Ns levels (ΔDiHS-NS ([ΔHexUA-GlcN(2-*N*-sulfate)). (**c**) Heparan sulfate Hs-0s levels (ΔDiHS-0S ([ΔHexUA-GlcNAc])). **—*p* < 0.001.

A total of 24 Ecuadorian patients were diagnosed with Sanfilippo syndrome type IIIB by the MPS Brazil Network, being 14 females (*n* = 58%) and 10 males (*n* = 42%) (Table 1). All patients were identified in the Manabí region, a Western province of Ecuador, or in neighboring regions (Figure 1). Manabí region has an area of approximately 19,000 km^2^ with estimated 1,593,000 inhabitants, which resulted in an incidence of MPS IIIB of 1.5:100,000 live births. In the patients with available data, mean age at diagnosis of patients was 8.8 y.o. (range 3–20 y.o., *n* = 15), while the mean age at onset of symptoms was 4.5 y.o. (range 0.5–12 y.o., *n* = 10), with an average diagnostic delay of 4.3 years (*p* > 0.005, *n* = 10) (Figure 2a).

### 3.2. Biochemical and Genetic Characterization

**Figure 3:**
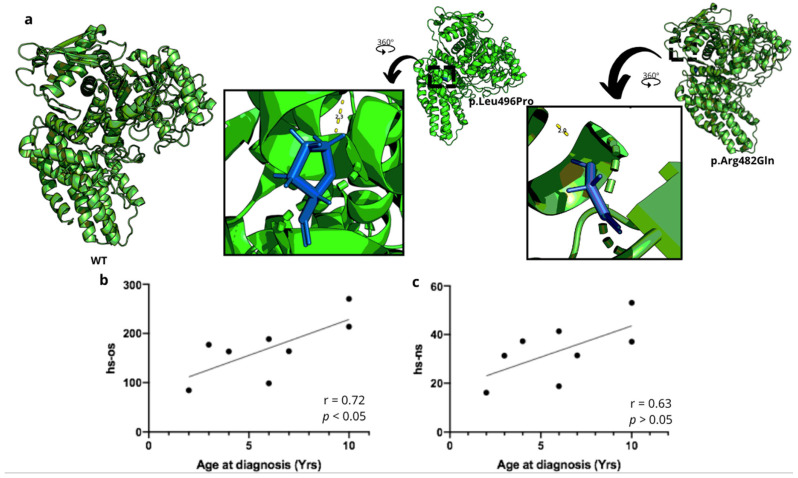
(**a**) Three-dimensional representation of pathogenic variants in MPS IIIB patients. (**b**) Correlation between Hs-0s values and age at diagnosis. (**c**) Correlation between Hs-Ns values and age at diagnosis.

Patients had elevated levels of urinary GAGs, specifically heparan sulfate. The heparan sulfate disaccharides (HS-NS and HS-OS) were significantly increased compared to reference values (Figure 2b,c). Enzyme activity was undetectable in all patients. The pathogenic variant c.1487T>C (p.Leu496Pro) was found in homozygosity in most patients, with one patient showing compound heterozygosity with the pathogenic variant c.1445G>A (p.Arg482Gln, indicated in Figure 1) and suggesting a possible founder effect. Three-dimensional analysis of the protein did not show significant structural changes (Figure 3a), but further protein-ligand characterizations are needed for future analyses. A positive correlation was found between the age of diagnosis and the concentration of HS-OS, but not with HS-NS (Figure 3b,c). 

### 3.3. Clinical Characterization

The age of onset for each symptom is detailed in Supplementary Table S1. The mean age for loss of walking ability was approximately 10.3 ± 2.51 years old, and for speech ability was around 6.4 ± 2.95 years old (Table 2). Interestingly, in this sample of Ecuadorian MPS IIIB patients, males exhibited more severe symptoms compared to females, particularly in the age of onset of neurological manifestations. The primary clinical characteristic observed in most Ecuadorian patients was neuro-psychomotor developmental delay (9 out of 10), which is typical for MPS IIIB. Other common clinical presentations included neurological regression (65%), hepatomegaly (55%), growth retardation (50%), coarse facies (45%), and hernia (40%) (Table 3). Additional symptoms included heart disease (20%), upper respiratory tract infection (10%), hearing loss (5%), dysostosis multiplex, gibbosity, respiratory distress, and splenomegaly (5% each). 

### 3.4. mtDNA Analysis

**Figure 4:**
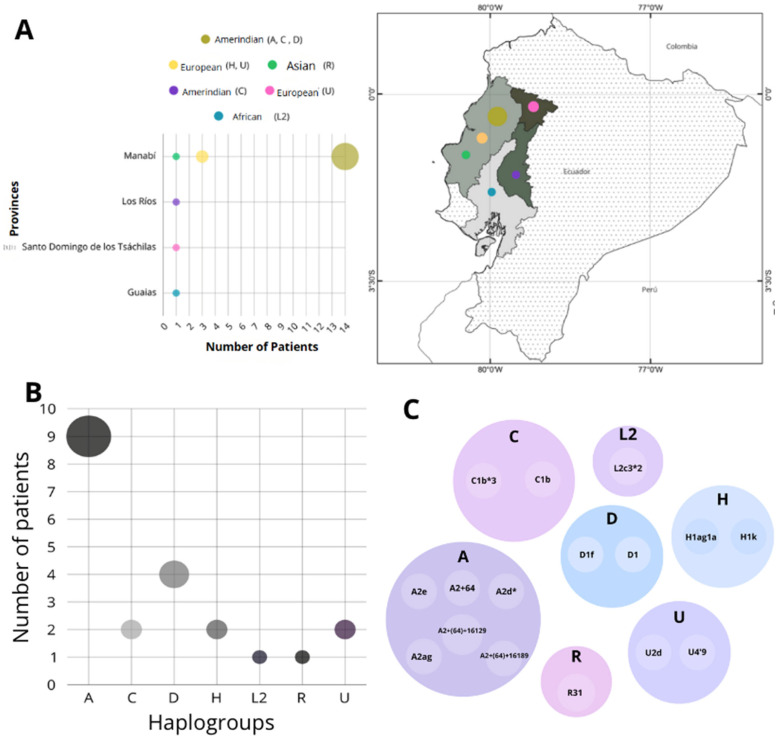
(**A**) Distribution of haplotypes among provinces. (**B**) Haplogroup frequencies. (**C**) Representation of sub-haplogroups.

After sequencing and performing individual sequence analysis on 21 patients, seven main haplogroups were identified, with 42.9% classified as A, 19% as D, while C, H, and U accounted for 9.5% each, and L2 and R each represented 4.8% (Figure 4A). Sub haplogroup A2 predominated in haplogroup A, as did C1b in C, and D1 in D. Haplogroup H, all cases belonged to H1, while L2c3 was the only variant detected in L2, and R31 in R. Haplogroup U, presented two distinct subgroups: U2 and U4 (Figure 4B). Regarding ancestral origin, haplogroups A, C, and D are associated with Amerindian lineage, while H and U are associated with European ancestry. Haplogroup R is linked to Asian roots, and L2 represents African heritage (Figure 4C). Predominant composition is Amerindian origin, corresponding to 71.4% of the total. “Haplogroup” and “province of origin” account for more than 80% of the genetic variability identified in the sample of 21 patients, with a strong statistically significant correlation (r = 0.466; *p* < 0.05). 

### 3.5. Symptom Heterogeneity and Maternal Ancestry

**Figure 5:**
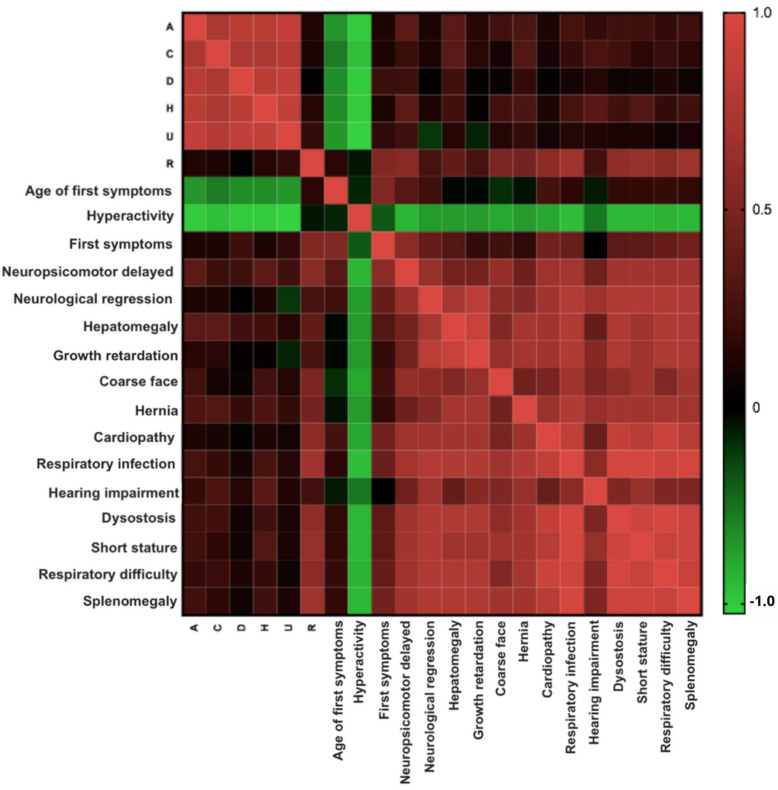
Multivariate analysis between data from MPS IIIB patients. Positive correlations are represented in red. Negative correlations are represented in green. Black represents zero correlations.

After conducting multivariate analysis and examining the data collected in this study, a correlation was found between the symptomatic presentation and the ancestry of the individuals. This suggests a potential mechanism that could help explain the varied symptomatic presentation in MPS IIIB (Figure 5).

## 4. Discussion

In the Manabí province the prevalence of MPS IIIB is 7.5 times higher than the global average (0.2 cases per 100,000 inhabitants) [1]. One of the probable reasons relates to consanguinity/endogamy, linked to cultural and historical characteristics present in relatively isolated populations [19,20,21].

All patients were originated from four provinces located in the Western region of Ecuador (Figure 4A), 85.6% of them coming from Manabí and 14.4% distributed among the neighboring provinces of Guayas, Los Ríos, and Santo Domingo de los Tsáchilas. Regarding gender distribution, female predominance was observed (58%), with 90.9% of these women belonging to Manabí. Meanwhile, men corresponded to 42 % of the total, of which 80% were residents of the same province. A relevant fact about the individuals is that, with exception of one compound heterozygous, they all possess the same homozygous pathogenic variant in the *NAGLU* gene, c.1487T>C p.(Leu496Pro), indicating a possible founder effect, exacerbated by endogamy/consanguinity. The founder effect is suspected, as the population exhibits a single shared mutation, is geographically clustered within a specific region of the country, and shows a predominance of shared maternal ancestry [18]. Nevertheless, confirmation of the founder effect requires the application of specific methodologies and further research. 

Clinical manifestations in the MPS IIIB patients closely correspond to the pattern described in other world populations [5,22]. Higher incidence of neurological manifestations in male patients, when compared to female patients, was not previously reported in the literature, to the best of our knowledge. Somatic manifestations, such as hepatomegaly, are less frequent. Limited access to health care and low socioeconomic conditions, as occurs in parts of Colombia, the Dominican Republic, and rural Brazil [23,24] were common, indicating one important factor to explains the relatively high age at diagnosis on these patients. 

Pathogenic variants in the *NAGLU* show a high degree of heterogeneity around the world [20,21], as reported in Turkey [25] and China [26]. On the other hand, the sample currently reported is homogeneous, being the p.Leu496Pro variant found predominantly in South America [1] and in areas which were exposed to European colonization [24].

Genetic investigation based on mitochondrial DNA from the Ecuadorian cluster demonstrated a significant geographic and ancestral concentration of the variant, particularly in the coastal region of Ecuador in province of Manabí. Female predominance and Amerindian heritage in association with mutation and kinship ties, indicate a likely founder effect [27]. As demonstrated by Slatki [28], founder effect is probably related to historical events/cultural traditions such as population bottlenecks and reduced genetic diversity. Rozenberg & Pereira [29] reinforced the role of founder with one single mutation in the population. Our findings are corroborated by Santos-Lopes et al. [15] with Brazilian patients with MPS IVA in the Northeast, where the prevalence of European ancestry (both maternal and paternal) and the presence of the same pathogenic variant, rarely found outside Brazil (only two cases in Sri Lanka), evidenced a clear founder effect explained by colonization processes and the high rate of consanguinity in the region.

Native American haplogroups, along with their respective subhaplogroups, are reflecting the incidence to Amerindian matrilineal ancestry. Baeta et al. [30], examining two local populations mtDNA (Kichwa and Mestizo) confirmed the predominance of the same main haplogroups. Haplogroup A2, widely distributed in North America, are consistent with gradual migration of early peoples across the continent, as well as in mestizo populations, reflecting fundamental role of indigenous lineages in the historical formation not only of Ecuador but of several American nations [30,31]. Waorani, considered likely the last remaining ethnic group from the pre-Columbian period in the Ecuadorian Amazon revealed the presence of only two haplogroups, A and D, subhaplogroups A2 and D1, as our results [32]. These results are in line with our study, once low genetic diversity may be associated with sociocultural factors, including the group’s geographic and social isolation and parental consanguinity [32].

Haplogroup L2, a genetic marker of African origin that, according to scientific evidence, originated in East Africa approximately 60,000 years ago before dispersing throughout Eurasia. Genetic studies have repeatedly identified African lineages as L2 in Ecuadorian population [33,34,35]. Among the identified haplogroups, H and U, both of European origin, each represented 9.5% of the analyzed sample. Haplogroup H, which constitutes the predominant European lineage, has a wide geographic distribution, encompassing not only Europe but also areas of Central, Southern, and Western Asia, Siberia, and northern Africa [36]. Haplogroup U, although, present in the European population characteristic of the Near East and North Africa [37]. Archaeogenetic research conducted by Keyser et al. [38], analyzed human remains unearthed in southern Siberia with the aim of reconstructing the initial migration patterns of Eurasian steppe populations. Analysis identified the presence of haplogroups U and H, including their respective subtypes, traditionally associated with Western Eurasia. Haplogroup R, associated with Asian ancestry, was identified in 4.8% of cases and is most prevalent in southern Asia [39]. Individual with this haplogroup presented subtype R31, which, according to studies such as that by Palanichamy et al. [40], originated in the Indian subcontinent. The main R lineage is estimated to have established itself in the region approximately 65,000 years ago [41]. Our findings corroborate with historical background of the Ecuadorian country.

## 5. Conclusions

In this study, we identified and characterized a cluster of MPS IIIB patients in Ecuador. This infor-mation can be useful to create specific strategies to this area, including genetic counseling. Male pa-tients exhibited more severe neurological symptoms compared to female patients (Supplementary Table S1), despite both genders carrying the same homozygous mutation. Furthermore, we observed a correlation between symptom presentation and ancestry. This correlation has become increasingly important in determining clinical characteristics in populations of patients with genetic diseases. The Amerindian ancestry observed in our sample showed a stronger correlation with symptomatic presentation. In particular, there seems to be a correlation between allelic presentation and ancestral origin, influencing the development of new technologies for classifying pathogenic variants, high-lighting the need for further research to explore this association. We highlight the importance of un-derstanding ancestry regarding clinical symptom presentation, especially its heterogeneity in rare genetic diseases. The establishment of cluster studies may be instrumental in better understanding and providing appropriate assistance to special populations with a high incidence of specific rare diseases.

## Figures and Tables

**Table 1:** Patients from Ecuador with Mucopolysaccharidosis type IIIB diagnosed in the present study. The trace means the information was not available.

No.	Gender	Current Age (Yrs)	Age at Diagnosis (Yrs)	NAGLU Activity (nmol/17 h/mg)	Urinary GAG (μg/mg Creatine) Disaccharide Method					Affected Family Members	DNA Variants	Parents Consanguinity	Reported Age of Onset of Symptoms (Yrs)	Initial Symptoms	Age (Yrs) 1st Seizure	Behavioral Problems	Current Status
					ds	mono-ks	hs-0s	hs-ns	di-ks								
1	M	13	6.3	Undetectable	-	-	-	-	-	-	c.1487T>C p.(Leu496Pro)/c.1487T>C p.(Leu496Pro)/	Second cousins	5	Neuropsychomotor developmental delay	-	-	Alive
2	F	20	12	Undetectable	58.44	83.80	98.55	18.76	5.83	-	c.1487T>C p.(Leu496Pro)/c.1487T>C p.(Leu496Pro)/	Third cousins	0.5	Hernia, hearing loss	8		Alive
3	F	-	-	Undetectable	-	-	-	-	-	-	c.1487T>C p.(Leu496Pro)/c.1487T>C p.(Leu496Pro)/	-	-	-	-	-	Alive
4	M	20	12	Undetectable	135.89	174.05	206.66	38.34	32.04	-	c.1487T>C p.(Leu496Pro)/c.1487T>C p.(Leu496Pro)/	-	1.5	Neuropsychomotor developmental delay	-	-	Alive
5	F	15	2	Undetectable	-	-	-	-	-	Brother	c.1487T>C p.(Leu496Pro)/c.1487T>C p.(Leu496Pro)/	-	-	-	-	-	-
6	M	17	4	Undetectable	70.36	46.27	84.39	16.10	7.56	Sister	c.1487T>C p.(Leu496Pro)/c.1487T>C p.(Leu496Pro)/	-	-	-	-	-	-
7	F	13	6	Undetectable	62.52	30.91	163.37	37.25	4.78	Brother	c.1487T>C p.(Leu496Pro)/c.1487T>C p.(Leu496Pro)/	First cousins	-	-	-	Hyperactivity	-
8	M	17	10	Undetectable	63.59	6.04	188.73	41.35	3.72	Sister	c.1487T>C p.(Leu496Pro)/c.1487T>C p.(Leu496Pro)/	First cousins	-	-	-	-	-
9	M	24	17	Undetectable	54.07	305.15	270.43	53.09	119.13	-	c.1487T>C p.(Leu496Pro)/c.1487T>C p.(Leu496Pro)/	-	2	Neuropsychomotor developmental delay	-	-	Alive
10	M	20	2	Undetectable	-	-	-	-	-	-	c.1487T>C p.(Leu496Pro)/c.1487T>C p.(Leu496Pro)/	-	-	-	-	-	Alive
11	M	17	10	Undetectable	-	-	-	-	-	-	c.1487T>C p.(Leu496Pro)/c.1487T>C p.(Leu496Pro)/	-	8	Neuropsychomotor developmental delay	10	-	-
12	F	-	-	Undetectable	76.14	140.48	213.88	37.09	31.08	-	c.1487T>C p.(Leu496Pro)/c.1487T>C p.(Leu496Pro)/	-	-		-	-	-
13	F	10	3.1	Undetectable	68.56	191.52	261.31	53.37	93.51	-	c.1487T>C p.(Leu496Pro)/c.1487T>C p.(Leu496Pro)/	-	3.1	Neuropsychomotor developmental delay	-	-	Alive
14	M	26	20	Undetectable	101.78	27.96	177.22	31.33	8.06	-	c.1487T>C p.(Leu496Pro)/c.1487T>C p.(Leu496Pro)/	-	5	Neuropsychomotor developmental delay	-	-	Alive
15	F	24	7	Undetectable	-	-	-	-	-	-	c.1487T>C p.(Leu496Pro)/c.1487T>C p.(Leu496Pro)/	First cousins	4	Neuropsychomotor developmental delay	-	-	Alive
16	F	-	-	Undetectable	74.70	23.46	163.75	31.44	3.65	-	c.1487T>C p.(Leu496Pro)/c.1487T>C p.(Leu496Pro)/		-				Alive
17	F	24	7	Undetectable	-	-	-	-	-	-	c.1487T>C p.(Leu496Pro)/c.1487T>C p.(Leu496Pro)/	-	4	Neuropsychomotor developmental delay	-	-	Alive
18	F	-	-	Undetectable	-	-	-	-	-	-	c.1487T>C p.(Leu496Pro)/c.1487T>C p.(Leu496Pro)/	-	-	-	-	-	Alive
19	F	11	-	Undetectable	116.23	56.80	117.08	24.86	11.01	-	c.1487T>C p.(Leu496Pro)/c.1487T>C p.(Leu496Pro)/	Second cousins	-	-	-		Alive
20	M	22	14	Undetectable	-	-	-	-	-	-	c.1487T>C p.(Leu496Pro)/c.1487T>C p.(Leu496Pro)/	-	12	Neuropsychomotor developmental delay	Present	14	Alive
21	F	17	-	Undetectable	-	-	-	-	-	-	c.1487T>C p.(Leu496Pro)/c.1487T>C p.(Leu496Pro)/	-	-	-	-	-	-
22	F	18	-	Undetectable	-	-	-	-	-	-	c.1487T>C p.(Leu496Pro)/c.1487T>C p.(Leu496Pro)/	-	-	-	-	-	-
23	M	-	-	Undetectable	-	-	-	-	-	-	c.1487T>C p.(Leu496Pro)/c.1487T>C p.(Leu496Pro)/	-	-	-	-	-	-
24	M	-	-	Undetectable	-	-	-	-	-	-	c.1487T>C p.(Leu496Pro)/c.1445G>A (p.Arg482Gln)	-	-	-	-	-	-

**Table 2:** Developmental milestones in patients with Mucopolysaccharidosis type IIIB from Ecuador.

Neuropsychomotor Developmental Milestone	Age	
	Mean	SD
Sitting without support		
Acquisition (months) (n = 1)	16	-
Loss	-	-
Walking		
Acquisition (months) (n = 11)	18	5.67
Loss (years) (n = 3)	10.3	2.51
Speaking two-syllable words		
Acquisition (months) (n = 8)	16	11.61
Loss (years) (n = 0)	-	-
Two-word phrases		
Acquisition (months) (n = 5)	26.40	12.44
Loss (years) (n = 10)	6.4	2.95

**Table 3:** Clinical presentation of Ecuadorian patients with Mucopolysaccharidosis type IIIB. The last column indicates prevalence of that symptom, considering both genders. * Although we had 14 female patients in total, 4 had no data available and were not included in this analysis.

Clinical Symptom	Male Patients (10)	Female Patients (10 *)	Prevalence of Symptom in Patients (%)
Neuropsychomotor developmental delay	9	6	75%
Neurological regression	9	4	65%
Hepatomegaly	7	4	55%
Growth retardation	6	4	50%
Coarse face	5	4	45%
Hernia	4	4	40%
Heart disease/Valvulopathy	1	3	20%
Upper respiratory tract infection	2	0	10%
Hearing impairment	1	0	5%
Dysostosis multiplex	1	0	5%
Gibbosity	1	0	5%
Respiratory difficulty	1	0	5%
Splenomegaly	1	0	5%

## Data Availability

The data presented in this study are available on request from the corresponding author.

## Supplementary Materials

The following supporting information can be downloaded at https://www.mdpi.com/article/10.3390/diagnostics15182337/s1, Table S1: Clinical presentation in male and female patients.
