# Supplementary material for: Comprehensive Characterization of a Cluster of Mucopolysaccharidosis IIIB in Ecuador
Source: Diagnostics (Basel). 2025 Sep 15;15(18):2337. doi: 10.3390/diagnostics15182337 (PMC12468815; doi:10.3390/diagnostics15182337)
Supplement: Supplementary file 1 [file diagnostics-15-02337-s001.zip › diagnostics-3834009-supplementary.pdf]

Supplementary Table S1: Clinical presentation in male and female patients.

| Age Interval (cumulative) | Neuropsychomotor Development Delay | | Neurological Regression | | Growth Delay | | Hepatomegaly | | Hernia | | Infiltrated Face | | Hearing Impairment | | Upper Respiratory Tract Infection | | Heart disease/ Valvulopathy | |
|---|---|---|---|---|---|---|---|---|---|---|---|---|---|---|---|---|---|---|
| | **Male (n)** | **Female (n)** | **Male (n)** | **Female (n)** | **Male (n)** | **Female (n)** | **Male (n)** | **Female (n)** | **Male (n)** | **Female (n)** | **Male (n)** | **Female (n)** | **Male (n)** | **Female (n)** | **Male (n)** | **Female (n)** | **Male (n)** | **Female (n)** |
| 0-1 | 0 | 0 | 0 | 0 | 0 | 0 | 0 | 0 | 0 | 0 | 0 | 0 | 0 | 0 | 0 | 0 | 0 | 0 |
| 0-4 | 3 | 1 | 3 | 0 | 2 | 0 | 2 | 0 | 1 | 1 | 2 | 0 | 0 | 0 | 2 | 0 | 0 | 0 |
| 0-7 | 4 | 4 | 4 | 3 | 3 | 3 | 3 | 2 | 2 | 2 | 3 | 2 | 0 | 1 | 2 | 0 | 0 | 1 |
| 0-10 | 5 | 4 | 5 | 4 | 4 | 3 | 4 | 2 | 2 | 2 | 3 | 3 | 0 | 0 | 2 | 0 | 1 | 2 |
| 0-13 | 6 | 6 | 6 | 4 | 4 | 4 | 4 | 4 | 3 | 4 | 3 | 4 | 0 | 0 | 2 | 0 | 1 | 2 |
| 0-16 | 7 | 6 | 7 | 4 | 5 | 4 | 5 | 4 | 3 | 4 | 4 | 4 | 0 | 0 | 2 | 0 | 1 | 2 |
| 0-19 | 8 | 6 | 8 | 4 | 6 | 4 | 6 | 4 | 3 | 4 | 5 | 4 | 0 | 0 | 2 | 0 | 1 | 3 |
| 0-22 | 9 | 6 | 9 | 4 | 6 | 4 | 7 | 4 | 4 | 4 | 5 | 4 | 1 | 0 | 2 | 0 | 1 | 3 |
| Total of Patients (%) | 75% | | 65% | | 50% | | 55% | | 40% | | 45% | | 5% | | 10% | | 20% | |
